# Ambient ammonia synthesis via palladium-catalyzed electrohydrogenation of dinitrogen at low overpotential

**DOI:** 10.1038/s41467-018-04213-9

**Published:** 2018-05-15

**Authors:** Jun Wang, Liang Yu, Lin Hu, Gang Chen, Hongliang Xin, Xiaofeng Feng

**Affiliations:** 1 Department of Physics, University of Central Florida, Orlando, FL 32816 USA; 2 Department of Chemical Engineering, Virginia Polytechnic Institute and State University, Blacksburg, VA 24061 USA; 3 Department of Materials Science and Engineering, University of Central Florida, Orlando, FL 32816 USA; 4 Department of Chemistry, University of Central Florida, Orlando, FL 32816 USA

## Abstract

Electrochemical reduction of N_2_ to NH_3_ provides an alternative to the Haber−Bosch process for sustainable, distributed production of NH_3_ when powered by renewable electricity. However, the development of such process has been impeded by the lack of efficient electrocatalysts for N_2_ reduction. Here we report efficient electroreduction of N_2_ to NH_3_ on palladium nanoparticles in phosphate buffer solution under ambient conditions, which exhibits high activity and selectivity with an NH_3_ yield rate of ~4.5 μg mg^−1^_Pd_ h^−1^ and a Faradaic efficiency of 8.2% at 0.1 V vs. the reversible hydrogen electrode (corresponding to a low overpotential of 56 mV), outperforming other catalysts including gold and platinum. Density functional theory calculations suggest that the unique activity of palladium originates from its balanced hydrogen evolution activity and the Grotthuss-like hydride transfer mechanism on α-palladium hydride that lowers the free energy barrier of N_2_ hydrogenation to *N_2_H, the rate-limiting step for NH_3_ electrosynthesis.

## Abstract (web summary)

The Haber-Bosch process, producing NH_3_ from N_2_, is a crucial yet energetically demanding reaction, inspiring interest in the exploration of ambient-condition alternatives. Here, authors develop a palladium electrocatalyst that shows a high selectivity and activity for N_2_ reduction to NH_3_.

## Introduction

Due to the limited supply of fossil fuels, there is a critical demand to use renewable energy to drive the chemical processes that have heavily relied on the consumption of fossil fuels^1^. One such energy-intensive chemical process is the Haber−Bosch process^2, 3^, which produces NH_3_ from N_2_ and H_2_ using iron-based catalysts under high temperature (350−550 °C) and high pressure (150−350 atm). NH_3_ is one of the most highly produced inorganic chemicals in the world, because of its vast need in fertilizer production, pharmaceutical production, and many other industrial processes^4, 5^. In 2015, around 146 million tons of NH_3_ was produced globally through the Haber−Bosch process^6^, which consumes 3−5% of the annual natural gas production worldwide, approximating to 1−2% of the global annual energy supply^2, 5^. This industrial process is also responsible for >1% of the global CO_2_ emissions^2^. Therefore, it is highly desirable to develop an alternative, efficient process for NH_3_ synthesis using renewable energy^7–11^, which can simultaneously reduce the CO_2_ emissions.

One alternative approach to the Haber−Bosch process is to use electrical energy to drive the NH_3_ synthesis reaction under ambient conditions^12–15^, which can reduce the need for high temperature and pressure, and, thereby lower the energy demand. When powered by electricity from renewable energy sources such as solar and wind, electrochemical synthesis of NH_3_ from N_2_ and H_2_O can facilitate sustainable, distributed production of NH_3_, as well as the storage of renewable energy in NH_3_ as a carbon-neutral liquid fuel^16–18^, owing to its high energy density (4.32 kWh L^−1^), high hydrogen content (17.6 wt%), and facile liquidation (boiling point: −33.3 °C at 1 atm). However, the development of the process has been impeded by the lack of efficient electrocatalysts for N_2_ reduction reaction (N_2_RR). Although electrochemical synthesis of NH_3_ has been demonstrated on various materials including Ru, Pt, Au, Fe, and Ni^19–31^, most of them showed low activity and selectivity (typically, Faradaic efficiency <1%) for NH_3_ production^26–31^. Therefore, major improvement in N_2_RR catalysts is essential for the development of low-temperature NH_3_ electrolyzers, which necessitates a better understanding and control of the catalytic materials and the reaction kinetics.

There are two major challenges associated with electrochemical NH_3_ synthesis in aqueous media^32, 33^. From the thermodynamic point of view, the splitting of the strong N≡N bond requires a reduction potential where the hydrogen evolution reaction (HER) readily occurs, leading to an extremely low Faradaic efficiency for N_2_RR under ambient conditions^26–31^. Therefore, it would be optimal to find a catalytic system that can promote N_2_RR at low overpotentials and suppress the competing HER. From the kinetic perspective, it is suggested that the rate-determining step for N_2_RR is the formation of *N_2_H through a proton-coupled electron transfer process (H^+^ + e^−^ + * + N_2_ → *N_2_H)^33, 34^, where * signifies an active site on the catalyst surface. It involves a proton from the electrolyte, an electron transferred from the electrode, and a N_2_ molecule in the solution. The strong solvent reorganization required for the endergonic charge transfer steps has a low probability of occurrence, leading to the sluggish kinetics. Instead, if the atomic *H species can be formed on a catalyst surface and directly react with N_2_, it may largely accelerate the kinetics to form *N_2_H: *H + N_2_ → *N_2_H. Indeed, there have been several investigations of metal hydride complexes for N_2_ reduction in a homogenous medium^35, 36^. Similarly, NH_3_ synthesis has been achieved at a low temperature by LiH-mediated N_2_ transfer and hydrogenation on the transition metals^37^. Therefore, it is imperative to examine such a hydrogenation pathway for the electrochemical N_2_RR in aqueous electrolyte systems under ambient conditions.

Here we report an ambient electrochemical reduction of N_2_ to NH_3_ on carbon black-supported Pd nanoparticles (Pd/C), which can form Pd hydrides under certain potentials and promote surface hydrogenation reactions. Operating in a N_2_-saturated phosphate buffer solution (PBS) electrolyte, the Pd/C catalyst enables NH_3_ production with a yield rate of around 4.5 μg mg^−1^_Pd_ h^−1^ and a high Faradaic efficiency of 8.2% at 0.1 V vs. the reversible hydrogen electrode (RHE), which corresponds to a low overpotential of 56 mV. This catalytic performance is enabled by an effective suppression of the HER activity in the neutral PBS electrolyte and the Grotthuss-like hydride transfer mechanism on α-PdH for N_2_ hydrogenation. All potentials reported in this study are with respect to the RHE scale.

## Results

### Synthesis and characterization of Pd/C catalyst

**Fig. 1:**
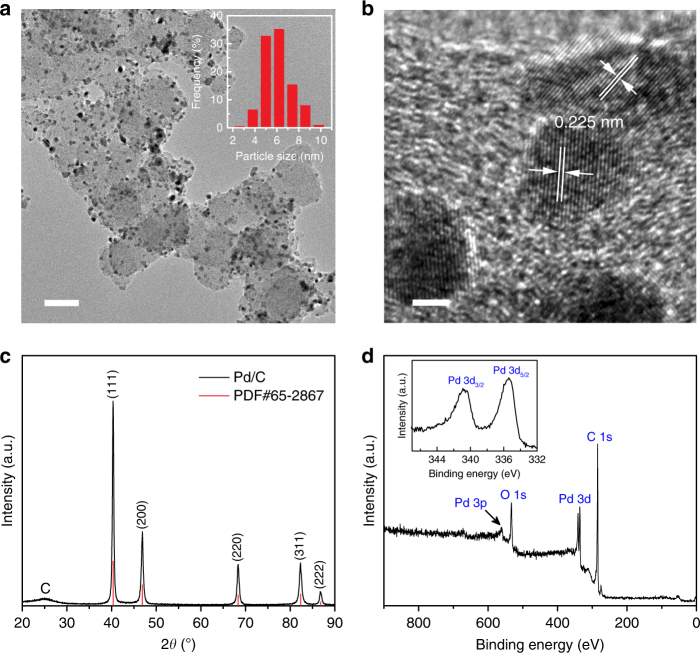
Structural and compositional characterizations of the Pd/C catalyst. **a** TEM and **b** high-resolution TEM images of the Pd/C catalyst. Inset **a**: particle size distribution. Scale bars, **a** 50 nm; **b** 2 nm. **c** XRD pattern and **d** XPS survey spectrum of the Pd/C catalyst. Inset **d**: high-resolution XPS spectrum of Pd 3d peaks

The Pd/C catalyst was prepared using polyol reduction method (see Methods section for experimental details). Figure 1a shows a representative transmission electron microscopy (TEM) image of the obtained Pd/C catalyst, which suggests that the Pd nanoparticles are homogeneously dispersed on the carbon black. The nanoparticle sizes have a narrow distribution between 4 and 9 nm, with an average size of around 6 nm (see the inset of Fig. 1a). A high-resolution TEM image in Fig. 1b shows the atomic lattice fringes of the particles with lattice plane spacings determined to be 0.225 nm, corresponding to the (111) lattice spacing of Pd. An X-ray diffraction (XRD) pattern of the Pd/C catalyst is shown in Fig. 1c, in which the peaks with 2*θ* values of 40.1^o^, 46.6^o^, 68.1^o^, 82.1^o^, and 86.6^o^ can be indexed to the diffraction from the (111), (200), (220), (311), and (222) lattice planes of Pd, respectively (PDF#65-2867). X-ray photoelectron spectroscopy (XPS) was used to examine the elemental composition of the Pd/C catalyst. As shown in Fig. 1d, only Pd, C, and O were observed in the survey spectrum, where the binding energies of 335.4 and 340.9 eV correspond to the 3d_5/2_ and 3d_3/2_ levels of metallic Pd^0^. Of note, no N species were observed within the detection limit of the XPS (~0.1 atomic percent), as shown in Supplementary Fig. 1.

### Electroreduction of N_2_ to NH_3_ on the Pd/C catalyst

The electrochemical measurements were performed using a gas-tight two-compartment electrochemical cell separated by a piece of Nafion 115 membrane (Supplementary Fig. 2). A piece of Pt gauze and Ag/AgCl electrode (filled with saturated KCl solution) were used as counter electrode and reference electrode, respectively. The working electrode was prepared by dispersing the Pd/C catalyst on a carbon paper or a glassy carbon substrate, as specified below. N_2_ gas was delivered into the cathodic compartment by N_2_ gas bubbling. The N_2_RR activities of the electrodes were evaluated using controlled potential electrolysis with N_2_-saturated electrolyte for 3 h. All potentials were iR-compensated and converted to the RHE scale via calibration (Supplementary Fig. 3). The gas-phase product (H_2_) was quantified by periodic gas chromatography of the headspace. The produced NH_3_ in the solution phase was quantified at the end of each electrolysis using the calibration curves established by the indophenol blue method^38^ (Supplementary Fig. 4). Another possible solution-phase product, N_2_H_4_, was also determined using a spectrophotometric method developed by Watt and Chrisp^39^ (Supplementary Fig. 5), whereas no N_2_H_4_ was detected in our studies within the detection limit of the method.

**Fig. 2:**
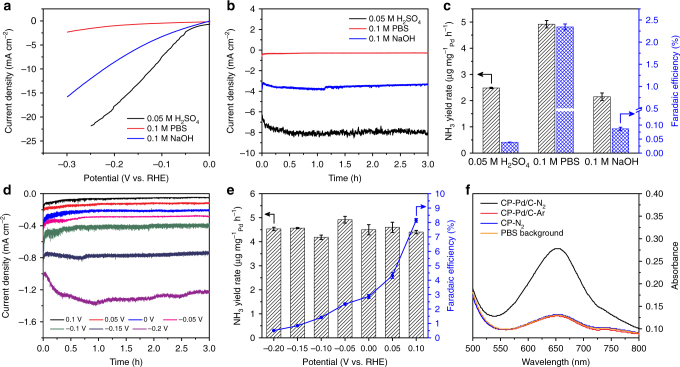
Electroreduction of N_2_ to NH_3_ on the Pd/C catalyst. **a** Linear sweep voltammetric curves of the Pd/C catalyst on the glassy-carbon support measured in the three Ar-saturated electrolytes. **b** Chronoamperometric curves of the Pd/C catalyst on the carbon-paper support measured at −0.05 V vs. RHE in the three N_2_-saturated electrolytes, and **c** corresponding NH_3_ yield rates and Faradaic efficiencies in the three electrolytes. **d** Chronoamperometric curves of the Pd/C catalyst on the carbon-paper support in N_2_-saturated 0.1 M PBS at various potentials, and **e** corresponding NH_3_ yield rates and Faradaic efficiencies at the potentials. The error bars correspond to the standard deviations of measurements over three separately prepared samples under the same conditions. **f** UV-vis absorption spectra of the electrolytes after electrolysis at −0.05 V vs. RHE for 3 h under different conditions. No apparent NH_3_ was detected for the control experiments with Ar-saturated electrolyte (CP-Pd/C-Ar) or without Pd catalyst (CP-N_2_), indicating that NH_3_ was produced by Pd-catalyzed electroreduction of N_2_

To boost the selectivity for N_2_RR, we need to find an electrolyte that can effectively suppress the competing HER. We compared the HER activities of the Pd/C catalyst in three Ar-saturated electrolytes: 0.05 M H_2_SO_4_ (pH = 1.2), 0.1 M PBS (pH = 7.2), and 0.1 M NaOH (pH = 12.9). Linear sweep voltammograms (LSV) of the Pd/C catalyst in the three electrolytes show that the current densities measured in H_2_SO_4_ and NaOH are both several times higher than those in PBS in a wide potential range (see Fig. 2a and Supplementary Fig. 6a), indicating an effective suppression of the HER activity in the neutral PBS electrolyte. The less favorable kinetics of HER in PBS is because of its higher barrier for mass- and charge-transfer^40^, as evidenced by the electrochemical impedance spectra in Supplementary Fig. 6b. Similarly, the controlled potential electrolysis in N_2_-saturated PBS at −0.05 V vs. RHE produces a current density of about 0.3 mA cm^−2^ (Fig. 2b), which is much lower than that in H_2_SO_4_ (~8 mA cm^−2^) and NaOH (~3.5 mA cm^−2^). However, the NH_3_ yield rate in PBS reaches 4.9 μg mg^−1^_Pd_ h^−1^ (Fig. 2c), which is around two times of that in H_2_SO_4_ (2.5 μg mg^−1^_Pd_ h^−1^) and that in NaOH (2.1 μg mg^−1^_Pd_ h^−1^). More strikingly, a Faradaic efficiency of 2.4% is achieved in PBS, whereas both H_2_SO_4_ and NaOH electrolytes give rise to a Faradaic efficiency lower than 0.1%. These results clearly indicate that PBS is a promising electrolyte for electrochemical N_2_RR due to its effective suppression of the HER activity. Therefore, all the following N_2_RR experiments were performed with 0.1 M PBS electrolyte.

Subsequently, the activities of the Pd/C catalyst for N_2_RR were systematically investigated in N_2_-saturated PBS at various potentials with separately prepared electrodes. As shown in Fig. 2d, the total current density increases from ~0.05 to more than 1.2 mA cm^−2^, as the potential shifts from 0.1 to −0.2 V. Interestingly, the NH_3_ yield rate remains similar within this potential range, fluctuating around 4.5 μg mg^−1^_Pd_ h^−1^ (Fig. 2e). Strikingly, the Faradaic efficiency for NH_3_ production reaches a maximum value of 8.2% at 0.1 V, which corresponds to a low overpotential of 56 mV, given the equilibrium potential of 0.156 V for N_2_ reduction to NH_3_ under our experimental conditions (see Methods section for the calculations). The Faradaic efficiency decreases gradually at more negative potentials, which is mainly caused by the rapid rising of the HER activity (Supplementary Fig. 7). To the best of our knowledge, the Pd/C catalyst achieves an NH_3_ yield rate and Faradaic efficiency that are comparable to the recently reported catalysts for N_2_RR under ambient conditions (Supplementary Table 1), but uses an overpotential lower by at least 300 mV, making it one of the most active and selective electrocatalysts for ambient NH_3_ synthesis.

In addition, we have carefully examined the N source of the produced NH_3_. First, control experiments with Ar-saturated electrolyte or without Pd catalyst were performed. As shown in Fig. 2f, no apparent NH_3_ was detected using the indophenol blue method when the bubbled N_2_ gas was replaced by Ar or when a carbon paper electrode without Pd was used, indicating that the NH_3_ was produced by N_2_ reduction in the presence of Pd catalyst. Furthermore, ^15^N isotopic labeling experiment was performed as an alternative method to verify the N source of the produced NH_3_ in 0.1 M PBS electrolyte. A triplet coupling for ^14^NH_4_^+^ and a doublet coupling for ^15^NH_4_^+^ in the ^1^H nuclear magnetic resonance (^1^H NMR) spectra are used to distinguish them^25^. As shown in Supplementary Fig. 8, only ^15^NH_4_^+^ was observed in the electrolyte when ^15^N_2_ was supplied as the feeding gas, and no NH_4_^+^ was detected when Ar was supplied, which are consistent with the control experiments and confirm that the NH_3_ was produced by Pd-catalyzed electroreduction of N_2_.

The stability of the Pd/C catalyst for electrochemical N_2_RR was assessed by consecutive recycling electrolysis at −0.05 V vs. RHE. After five consecutive cycles, only a slight decline in the total current density was observed, as shown in Supplementary Fig. 9. However, the NH_3_ yield rate and Faradaic efficiency decreased to 2.4 μg mg^−1^_Pd_ h^−1^ and 1.2% after five cycles, indicating a loss of the N_2_RR activity by 50% after the 15 h operation. The decrease in the N_2_RR activity is due to the loss of active Pd surface area caused by the aggregation of Pd nanoparticles on the carbon support, as evidenced by the TEM images of the Pd/C catalyst after the recycling test (Supplementary Fig. 9). Further improvement in the dispersion of Pd nanoparticles on the support and interaction between them will be beneficial for the long-term durability of the catalyst.

## Discussion

**Fig. 3:**
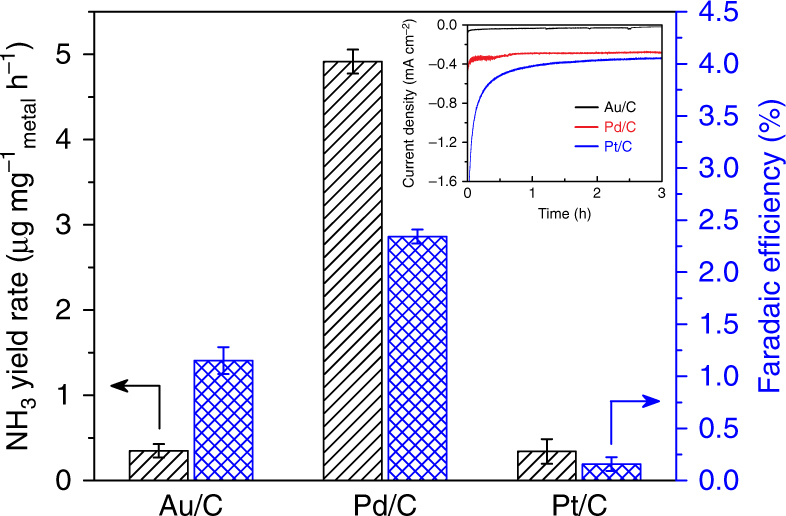
Comparison of the Pd/C catalyst with Au/C and Pt/C catalysts for N_2_RR. NH_3_ yield rates and Faradaic efficiencies of the Au/C, Pd/C, and Pt/C catalysts on the carbon-paper support measured in N_2_-saturated 0.1 M PBS electrolyte at −0.05 V vs. RHE. The error bars correspond to the standard deviations of measurements over three separately prepared samples under the same conditions. Inset: chronoamperometric curves obtained for each catalyst

The Pd/C catalyst exhibits high activity and selectivity for the N_2_RR at a low overpotential of 56 mV. To explore the underlying mechanism and to see whether it is exclusive to Pd, we prepared Au/C and Pt/C catalysts with identical metal loading using the same method, and compared their N_2_RR catalytic performance with that of the Pd/C catalyst (Fig. 3). The structural and compositional characterizations of the Au/C and Pt/C catalysts, including TEM, XRD, and XPS (Supplementary Fig. 10), confirm the successful synthesis of the metal nanoparticles with similar sizes as that of the Pd/C catalyst. At −0.05 V vs. RHE, the Au/C catalyst exhibits a current density (<0.04 mA cm^−2^) much lower than that of the Pd/C catalyst, whereas the Pt/C catalyst shows a slightly higher current density (see inset of Fig. 3). Both the Au/C and Pt/C catalysts produce NH_3_ at a rate of about 0.3 μg mg^−1^_metal_ h^−1^, which is lower than that of the Pd/C catalyst by more than one order of magnitude. The Au/C catalyst achieves a Faradaic efficiency of 1.2% for N_2_RR, due to its low activity for the HER. In contrast, the Faradaic efficiency for N_2_RR on the Pt/C catalyst is only 0.2%, much lower than that of the Au/C catalyst, because Pt is intrinsically the most active catalyst for HER (Supplementary Fig. 11). In comparison, such a significant difference in both N_2_RR activity and selectivity clearly indicates that Pd is a unique catalyst for N_2_RR. Actually, Pd can readily absorb H atoms in its lattice, forming Pd hydrides under operating conditions^41^. The cathodic current observed at 0.10 and 0.05 V is similar to the data in a previous study^42^, and the unaccounted current at the two potentials (see Supplementary Fig. 7b) may be similarly attributed to the dynamic hydrogen adsorption and absorption on Pd^42^, in addition to the capacitance of the carbon support. It has been reported that Pd catalyzes the electroreduction of CO_2_ to formate with high activity and selectivity at low overpotentials^43, 44^, and both experimental and computational studies have confirmed that it is attributed to a hydrogenation mechanism through in situ formed PdH_x_ phase^45^. Interestingly, a recent study of N_2_RR on commercial Pd/C catalysts in acidic and alkaline electrolytes showed Faradaic efficiencies lower than 0.1% at −0.2 V vs. RHE^31^, which are consistent with our results under similar conditions (Fig. 2c), and highlight the critical roles of the HER suppression in PBS and the hydrogenation via hydride transfer pathway at low overpotentials (vide infra).

**Fig. 4:**
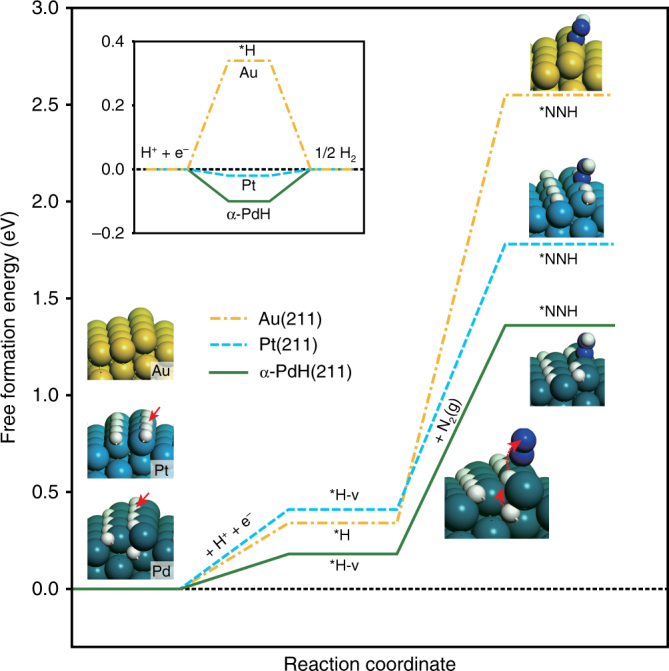
Computational studies. DFT-calculated free energy pathways of HER (inset) and the relevant steps of N_2_RR on the (211) surfaces of Au, Pt, and α-PdH at surface potential of 0 V vs. RHE under 298.15 K (atomic structures shown in the insets). The first step denotes the formation of *H on the Au surface and *H vacancy (*H-v) on Pt and α-PdH step sites. The second step is the direct surface hydrogenation of N_2_(g) forming *N_2_H on Au and Pt, and the Grotthuss-like hydride transfer pathway on α-PdH

To understand the unique activity of N_2_RR on Pd/C, we performed density functional theory (DFT) calculations for the energetics of HER and N_2_RR steps on the (211) surfaces of Au, Pt, and Pd hydride (see Supplementary Note 1 for the computational details). As employed previously, the undercoordinated step atoms are assumed to be the catalytic site for activating the N≡N bond^46^. For Pd, we have two subsurface *H (*H_sub_) at the octahedral sites underneath the Pd edge atoms to simulate the α-phase Pd hydride (α-PdH), which is the stable phase under operating potentials^45^. According to the differential adsorption free energies of *H on the (211) surfaces (Supplementary Fig. 12), we adopted models with 2/3 monolayer of *H for α-PdH and Pt, on which the step/terrace sites are occupied by *H and the bottom-of-the-edge sites are free, while a clean surface for Au was used (geometric structures shown in Fig. 4). In the inset of Fig. 4, we can see that the HER on Pt is facile with the free formation energy of *H close to zero^47^. In contrast, the process is limited by *H desorption on α-PdH and by *H adsorption on Au. Interestingly, the free energy cost in creating a *H vacancy (*H-v) necessary for N_2_ collision at step sites is much lower on α-PdH (0.18 eV) than that on Pt (0.41 eV). Furthermore, the hydrogenation of N_2_ by a surface *H to form *N_2_H on α-PdH (1.18 eV) is thermodynamically more favorable than that on Pt (1.37 eV) and Au (2.21 eV). On α-PdH, the surface hydrogenation is accompanied by the transfer of *H_sub_ to the surface site, analogous to the Grotthuss-like proton-hopping mechanism in a water network^48^. The reaction-free energy of N_2_ hydrogenation on Pd without the hydride transfer is less favorable by 0.3 eV (see Supplementary Fig. 13 for a direct comparison). The nature of this rate-determining step, i.e., surface chemical hydrogenation instead of the proton-coupled electron transfer, supports the observed weak potential dependence of the NH_3_ yield rate at low overpotentials. DFT-calculated free energies of hydrogenation of N_2_ vs. hydrogen evolution across metal surfaces (Au, Pt, and α-PdH) rationalize the activity and selectivity trends in Fig. 3.

In summary, we have discovered an efficient electrohydrogenation of N_2_ to NH_3_ on Pd/C catalyst at an overpotential as low as 56 mV for the electrochemical NH_3_ synthesis under ambient conditions. The Pd/C catalyst exhibits high activity and selectivity for N_2_RR in a PBS electrolyte, achieving an NH_3_ yield rate of about 4.5 μg mg^−1^_Pd_ h^−1^ and a Faradaic efficiency of 8.2% at 0.1 V vs. RHE. Comparative experiments indicate an effective suppression of the HER in the neutral PBS electrolyte, and a significantly higher N_2_RR activity of Pd than other catalysts including Au and Pt. The DFT calculations suggest that the in situ formed α-PdH allows the activation of N_2_ via a Grotthuss-like hydride transfer pathway that is thermodynamically more favorable than direct surface hydrogenation or proton-coupled electron transfer steps. Our findings open up an avenue to develop efficient electrocatalysts for not only the electroreduction of N_2_ to NH_3_, but also other challenging electrocatalytic reactions for renewable energy conversions.

## Methods

### Synthesis of carbon black-supported metal catalysts

The Pd/C catalyst was synthesized using polyol reduction method. First, the Pd precursor (K_2_PdCl_4_) solution was prepared by dissolving PdCl_2_ in water in the presence of KCl. Typically, 70 mg of carbon black was dispersed in 100 mL of ethylene glycol, followed by sonication for 30 min. Then, 5 mL of K_2_PdCl_4_ solution (Pd 6 mg mL^−1^) was added into the mixture. After stirring for another 30 min, the mixture was heated to 130 °C and kept at this temperature for 2 h. The catalyst slurry was filtered and washed with water. The resulting Pd/C catalyst was dried at 60 °C overnight, with a Pd loading of 30 wt%. Comparative samples of Au/C and Pt/C catalysts with a metal loading of 30 wt% were prepared using the same procedure, except with different metal precursors.

### Physical characterizations

TEM images were acquired using a FEI Tecnai F30 Transmission Electron Microscope, with a field emission gun operated at 200 kV. The XRD pattern was collected using a PANalytical Empyrean diffractometer, with a 1.8 KW copper X-ray tube. XPS was performed using a Physical Electronics 5400 ESCA photoelectron spectrometer. Gas products were analyzed by a gas chromatograph (SRI Multiple Gas Analyzer #5) equipped with molecular sieve 5 A and HayeSep D columns, with Ar as the carrier gas.

### Preparations of the working electrodes

First, 8 mg of carbon black-supported metal catalyst was dispersed in diluted Nafion alcohol solution containing 1.5 mL ethanol and 60 μL Nafion, which formed a homogeneous suspension after sonication for 1 h. Two types of substrates were used to prepare electrodes in this study: one was carbon paper, and the other was glassy carbon. The carbon-paper electrodes were prepared by drop-casting the suspension on a piece of carbon paper (1 × 1 cm^2^), with a total mass loading of 1 mg (of which 30 wt% is Pd), which were used for all controlled potential electrolyses. The glassy-carbon electrodes were prepared by drop-casting the suspension on a round glassy carbon (diameter = 3 mm), with a total mass loading of 0.435 mg cm^−2^ (of which 30 wt% is Pd), which were used for all linear sweep voltammetry measurements, except Supplementary Fig. 6a.

### Electrochemical measurements

Prior to N_2_RR tests, Nafion 115 membranes were heat-treated in 5% H_2_O_2_, 0.5 M H_2_SO_4_, and water for 1 h, respectively. After being rinsed in water thoroughly, the membranes were immersed in deionized water for future use. Electrochemical measurements were performed using a CH Instruments 760E Potentiostat, with a gas-tight two-compartment electrochemical cell separated by a piece of Nafion 115 membrane at room temperature (Supplementary Fig. 2). A piece of Pt gauze and Ag/AgCl/*sat*. KCl were used as counter electrode and reference electrode, respectively. The linear sweep voltammetry was scanned at a rate of 5 mV s^−1^. The N_2_RR activity of an electrode was evaluated using controlled potential electrolysis in an electrolyte for 3 h at room temperature (~293 K). Prior to each electrolysis, the electrolyte was presaturated with N_2_ by N_2_ gas bubbling for 30 min. During each electrolysis, the electrolyte was continuously bubbled with N_2_ at a flow rate of 10 sccm, and was agitated with a stirring bar at a stirring rate of ~800 rpm. No in-line acid trap was used to capture NH_3_ that might escape from the electrolyte in our study, as no apparent NH_3_ was detected in the acid trap under our experimental conditions. The applied potentials were iR-compensated, and the reported current densities were normalized to geometric surface areas.

### Calibration of the reference electrodes

All potentials in this study were converted to the RHE scale via calibration (Supplementary Fig. 3). The calibration was performed using Pt gauze as both working electrode and counter electrode in H_2_-saturated electrolyte. Cyclic voltammograms were acquired at a scan rate of 1 mV s^−1^. The two potentials at which the current equaled zero were averaged and used as the thermodynamic potential for the hydrogen electrode reactions.

### Ammonia quantification

The produced NH_3_ was quantitatively determined using the indophenol blue method^38^. Typically, 2 mL of the sample solution was first pipetted from the post-electrolysis electrolyte. Afterwards, 2 mL of a 1 M NaOH solution containing salicylic acid (5 wt%) and sodium citrate (5 wt%) was added, and 1 mL of NaClO solution (0.05 M) and 0.2 mL of sodium nitroferricyanide solution (1 wt%) were added subsequently. After 2 h, the absorption spectra of the resulting solution were acquired with an ultraviolet-visible (UV-vis) spectrophotometer (BioTek Synergy H1 Hybrid Multi-Mode Reader). The formed indophenol blue was measured by absorbance at *λ* = 653 nm. In order to quantify the produced NH_3_, the calibration curves were built using standard NH_4_Cl solutions in the presence of 0.05 M H_2_SO_4_, 0.1 M PBS, and 0.1 M NaOH, respectively (Supplementary Fig. 4), to take into account the possible influence of different pH values^38^. The measurements with the background solutions (no NH_3_) were performed for all experiments, and the background peak was subtracted from the measured peaks of N_2_RR experiments to calculate the NH_3_ concentrations and the Faradaic efficiencies.

### Hydrazine quantification

The yellow color developed upon the addition of *p*-dimethylaminobenzaldehyde (PDAB) to solutions of N_2_H_4_ in dilute hydrochloric acid solution was used as the basis for the spectrophotometric method to quantify the N_2_H_4_ concentration^39^. Typically, 5 mL of the electrolyte solution was taken out and then mixed with 5 mL of the coloring solution (4 g of PDAB dissolved in 20 mL of concentrated hydrochloric acid and 200 mL of ethanol). After 15 min, the absorption spectra of the resulting solution were acquired using a UV-vis spectrophotometer (BioTek Synergy H1 Hybrid Multi-Mode Reader). The solutions of N_2_H_4_ with known concentrations in 0.1 M PBS were used as calibration standards, and the absorbance at *λ* = 458 nm was used to plot the calibration curves (Supplementary Fig. 5).

### Calculation of the equilibrium potential

The standard potential for the half reaction of N_2_ reduction to NH_4_OH was calculated according to the standard molar Gibbs energy of formation at 298.15 K^49^. The equilibrium potential under our experimental conditions is calculated using the Nernst equation, assuming 1 atm of N_2_ and a NH_4_OH concentration of 0.01 mM in the solution.

1 $${\mathrm{N}}_{\mathrm{2}}\left( {\mathrm{g}} \right) + {\mathrm{2}}\,{\mathrm{H}}_{\mathrm{2}}{\mathrm{O}} + {\mathrm{6}}\,{\mathrm{H}}^{\mathrm{ + }} + {\mathrm{6}}\,{\mathrm{e}}^ - \to {\mathrm{2}}\,{\mathrm{NH}}_{\mathrm{4}}{\mathrm{OH}}\left( {{\mathrm{aq}}} \right)\,\,\,\,\,\,\,\,\,\Delta G^\circ = - 33.8\,{\mathrm{kJ}}\,{\mathrm{mol}}^{ - {\mathrm{1}}}$$

*E*° = −Δ*G*°/*nF* = 0.058 V, where *n* = 6 is the number of electrons transferred in the reaction and *F* is the Faraday constant.

The equilibrium potential under our experimental conditions is calculated using the Nernst equation, assuming 1 atm of N_2_ and a 0.01 mM concentration of NH_4_OH in the solution.

2 $$E = E^\circ - \frac{{RT}}{{6F}}\ln \left( {\frac{{\left[ {{\mathrm{NH}}_4{\mathrm{OH}}} \right]^2}}{{\left[ {{\mathrm{H}}^ + } \right]^6}}} \right) + 0.059{\mathrm{V}} \times {\mathrm{pH}} = 0.156{\mathrm{V}}\,{\mathrm{vs.}}\,{\mathrm{RHE}}$$

### Calculation of the Faradaic efficiency and the yield rate

The Faradaic efficiency was estimated from the charge consumed for NH_3_ production and the total charge passed through the electrode:

3 $${\mathrm{Faradaic}}\,{\mathrm{efficiency}} = (3F \times c_{{\mathrm{NH}}_{\mathrm{3}}} \times V)/Q$$

The yield rate of NH_3_ can be calculated as follows:

4 $${\mathrm{Yield}}\,{\mathrm{rate = }}(17c_{{\mathrm{NH}}_{\mathrm{3}}} \times V){\mathrm{/}}(t \times m)$$

where *F* is the Faraday constant (96,485 C mol^−1^), $c_{{\mathrm{NH}}_{\mathrm{3}}}$ is the measured NH_3_ concentration, *V* is the volume of the electrolyte, *Q* is the total charge passed through the electrode, *t* is the electrolysis time (3 h), and *m* is the metal mass of the catalyst (typically 0.3 mg). The reported NH_3_ yield rate, Faradaic efficiency, and error bars were determined based on the measurements of three separately prepared samples under the same conditions.

### ^15^N isotope labeling experiment

The isotopic labeling experiment was carried out using ^15^N_2_ as the feeding gas (Sigma-Aldrich, 98 atom % ^15^N) with 0.1 M PBS electrolyte. After electrolysis at −0.05 V vs. RHE for 10 h, 10 mL of the electrolyte was taken out and acidized to pH ~3 by adding 0.5 M H_2_SO_4_, and then concentrated to 2 mL by heating at 70 °C. Afterwards, 0.9 mL of the resulting solution was taken out and mixed with 0.1 mL D_2_O containing 100 ppm dimethyl sulphoxide (Sigma-Aldrich, 99.99%) as an internal standard for ^1^H nuclear magnetic resonance measurement (^1^H NMR, Bruker Avance III 400 MHz).

### Computational studies

DFT calculations were performed using the plane-wave-based PWSCF (Quantum-ESPRESSO) program and the Atomic Simulation Environment (ASE). The ultrasoft Vanderbilt pseudopotential method with Perdew–Burke–Ernzerhof (PBE) exchange-correlation functional was adopted. More calculation details and relevant references are provided in the Supplementary Note 1, Supplementary Table 2, and Supplementary References.

### Data availability

The data that support the findings of this study are available within the paper and its Supplementary Information file or are available from the corresponding authors upon reasonable request.

## Electronic supplementary material

Supplementary Information
