# Supplementary material for: Ambient ammonia synthesis via palladium-catalyzed electrohydrogenation of dinitrogen at low overpotential
Source: Nat Commun. 2018 May 15;9:1795. doi: 10.1038/s41467-018-04213-9 (PMC5953946; doi:10.1038/s41467-018-04213-9)
Supplement: Supplementary file 1 — Supplementary Information [file 41467_2018_4213_MOESM1_ESM.pdf]

Supplementary Information

Ambient ammonia synthesis via palladium-catalyzed electrohydrogenation of dinitrogen at low overpotential

Wang et al.

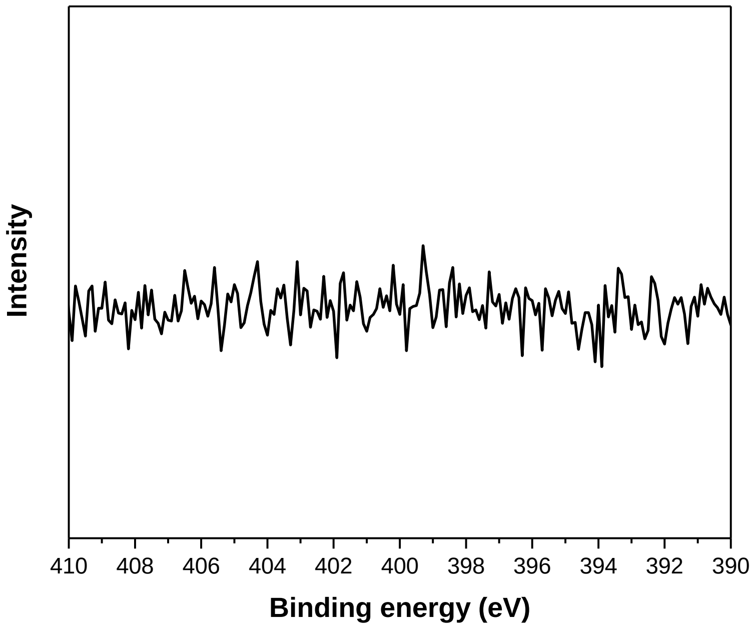

Supplementary Figure 1. High-resolution XPS spectrum of the Pd/C catalyst in the N 1s region. There are no distinguishable peaks in the region, indicating that no N species were observed within the detection limit of the XPS (~0.1 atomic percent).

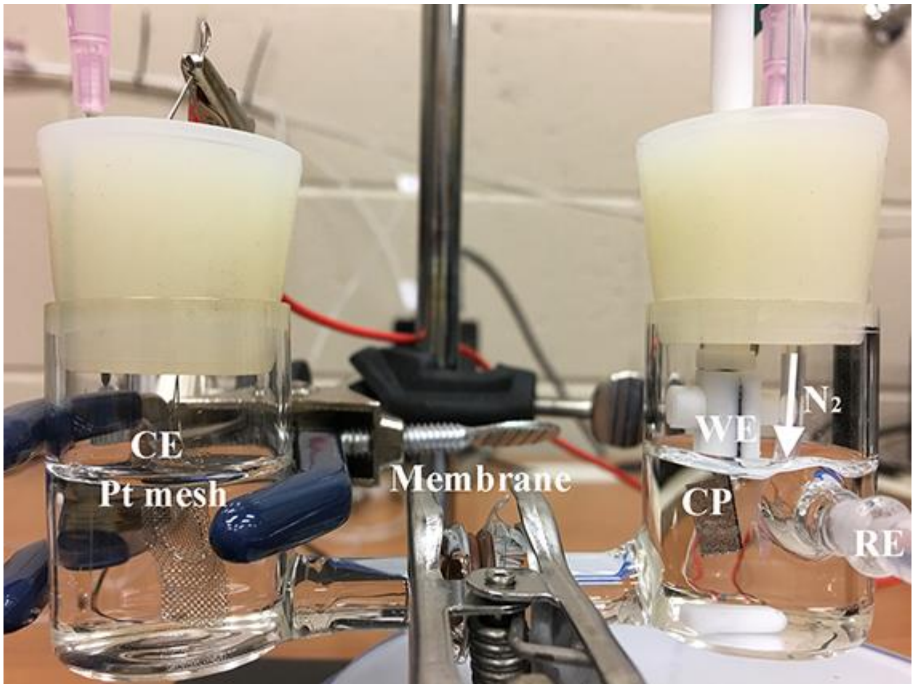

Supplementary Figure 2. Photograph of the electrochemical cell setup used for the N_2RR electrolysis.

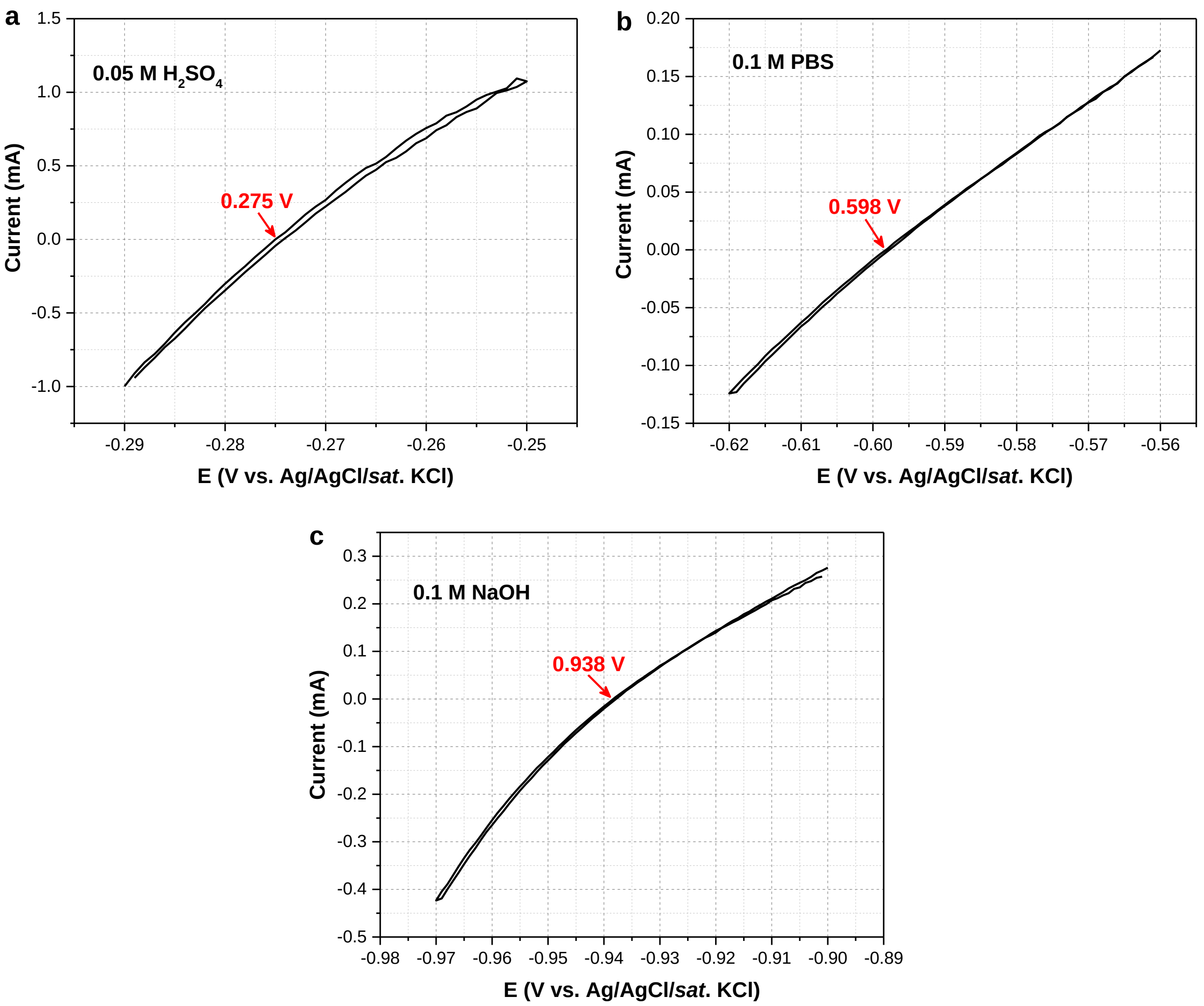

Supplementary Figure 3. Calibration of reference electrodes. The Ag/AgCl/saturated KCl reference electrode was calibrated with respect to RHE in **a** 0.05 M H_2SO_4, **b** 0.1 M PBS, and **c** 0.1 M NaOH, respectively. Based on the calibrations, we have:

in 0.05 M H_2SO_4, *E* (vs. RHE) = *E* (vs. Ag/AgCl) + 0.275 V;

in 0.1 M PBS, *E* (vs. RHE) = *E* (vs. Ag/AgCl) + 0.598 V;

in 0.1 M NaOH, *E* (vs. RHE) = *E* (vs. Ag/AgCl) + 0.938 V.

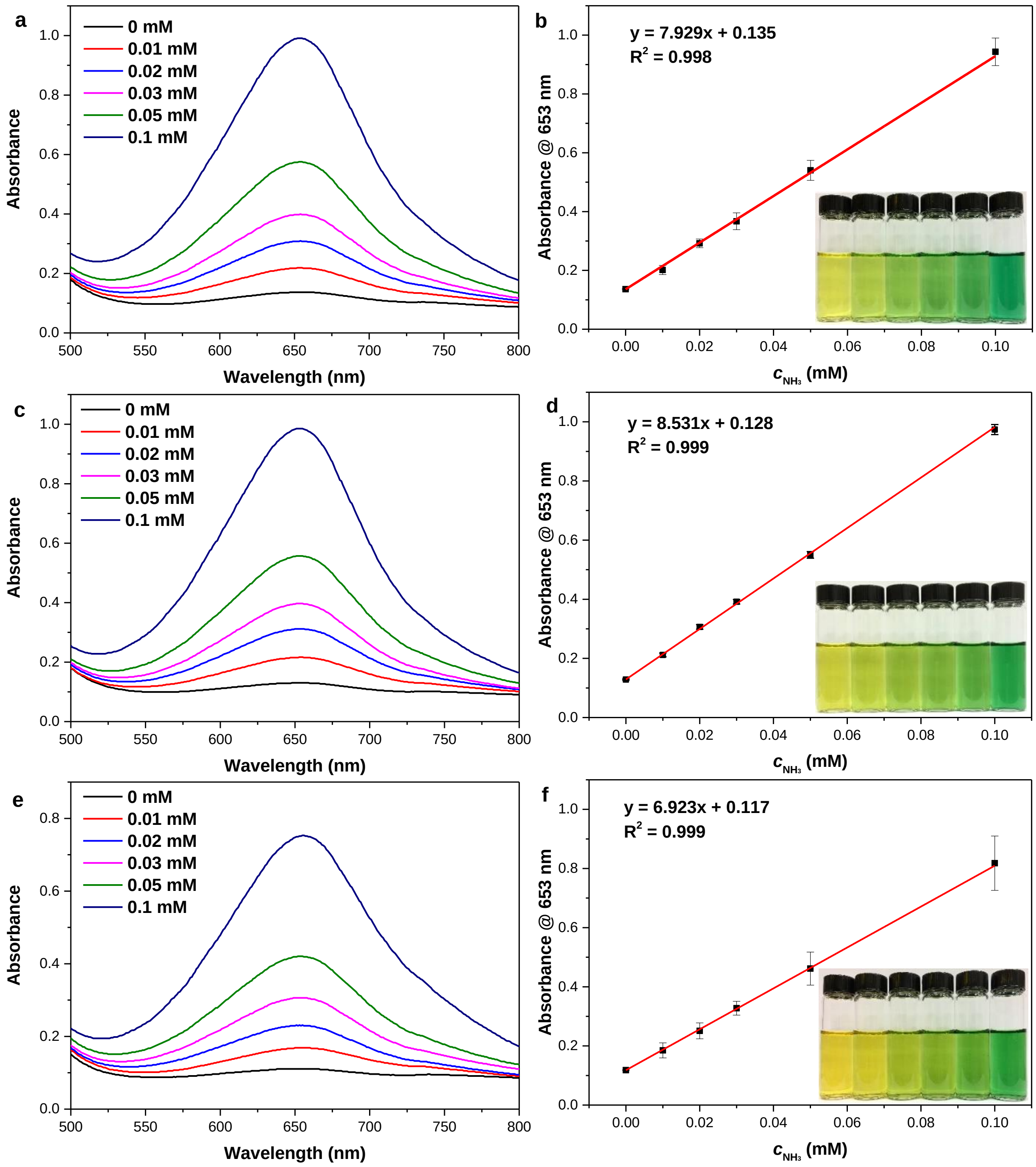

Supplementary Figure 4. NH_3 quantification using indophenol blue method. The UV-Vis absorption spectra and corresponding calibration curves for the colorimetric NH_3 assay using the indophenol blue method in different background solutions: **a, b** 0.05 M H_2SO_4, **c, d** 0.1 M PBS, and **e, f** 0.1 M NaOH. The error bars correspond to the standard deviations of multiple measurements.

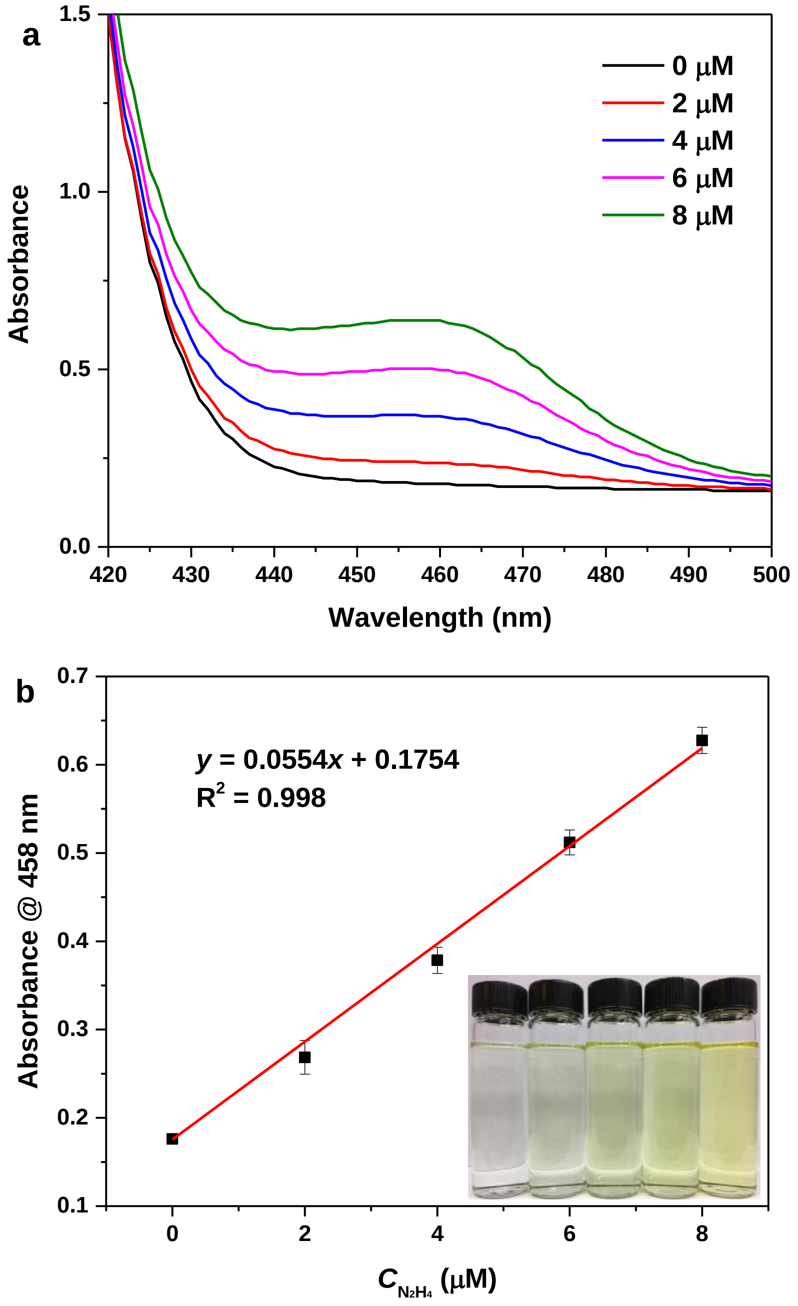

Supplementary Figure 5. N_2H_4 quantification. a The UV-Vis absorption spectra and **b** corresponding calibration curves for the colorimetric N_2H_4 assay in 0.1 M PBS. The error bars correspond to the standard deviations of multiple measurements.

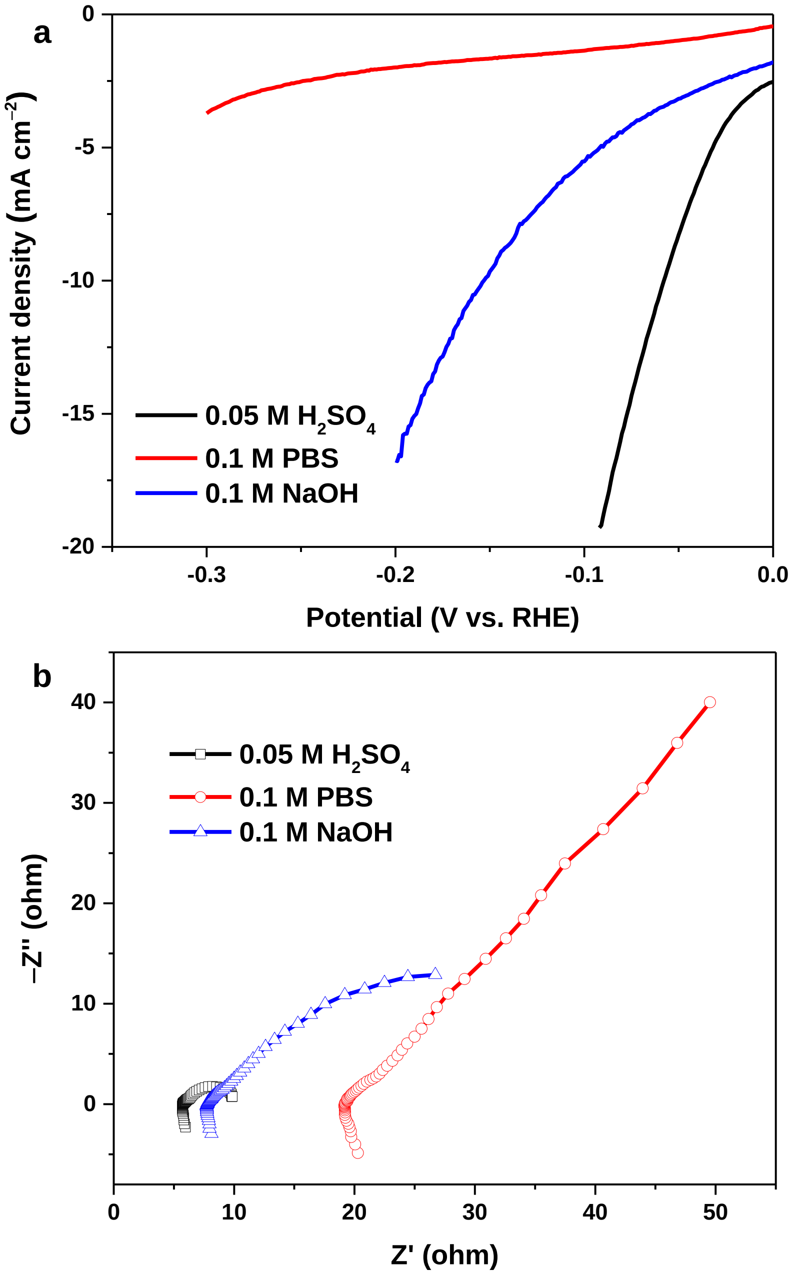

Supplementary Figure 6. Electrochemical measurements of carbon-paper-supported Pd/C catalyst. **a** Linear sweep voltammograms and **b** electrochemical impedance spectra of the carbon-paper-supported Pd/C catalyst (Pd/C loading = 1 mg cm^{-2}, with 30 wt % Pd) measured in three Ar-saturated electrolytes.

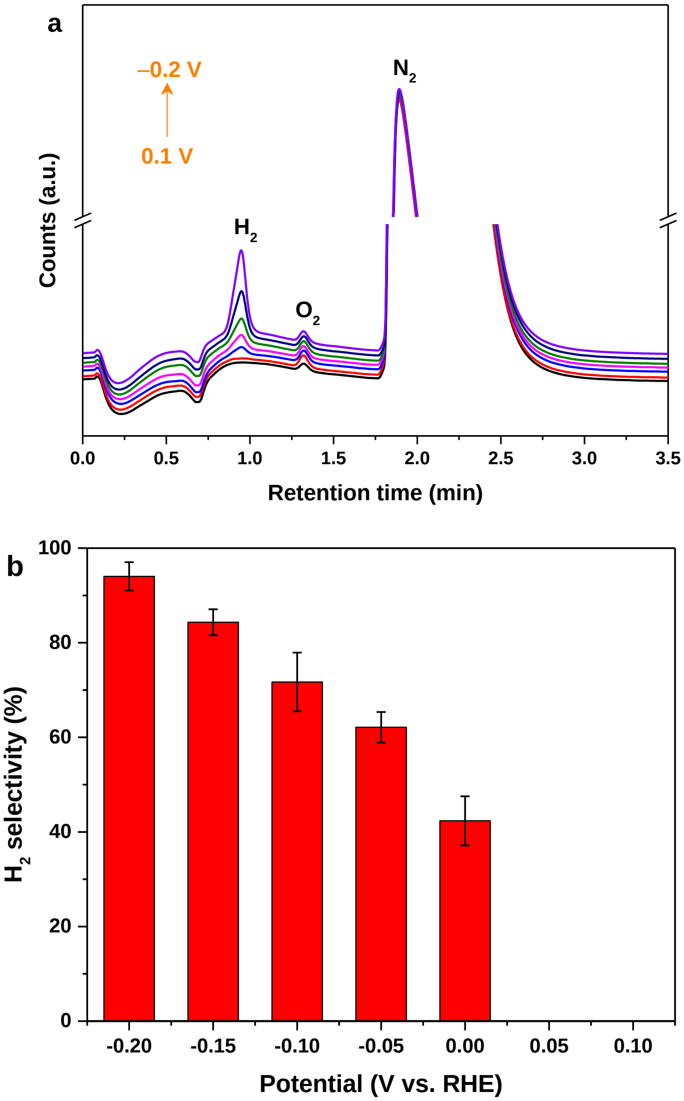

Supplementary Figure 7. Quantifying H_2 production at different potentials. a Gas chromatography (GC) spectra of the gas from the headspace of the cell for the N_2RR on the Pd/C catalyst in N_2-saturated 0.1 M PBS at various potentials, and **b** the calculated H_2 selectivity accordingly. The error bars correspond to standard deviations of measurements over three separately prepared samples under the same conditions. In the GC spectra, trace amount of residue oxygen was detected after long time N_2 flushing. H_2 signal was detected since 0 V and the H_2 selectivity increased rapidly at more negative potentials. Combing the data with the obtained NH_3 selectivity, the unaccounted value may be attributed to the capacitance of the carbon support as well as dynamic hydrogen adsorption and absorption on Pd.

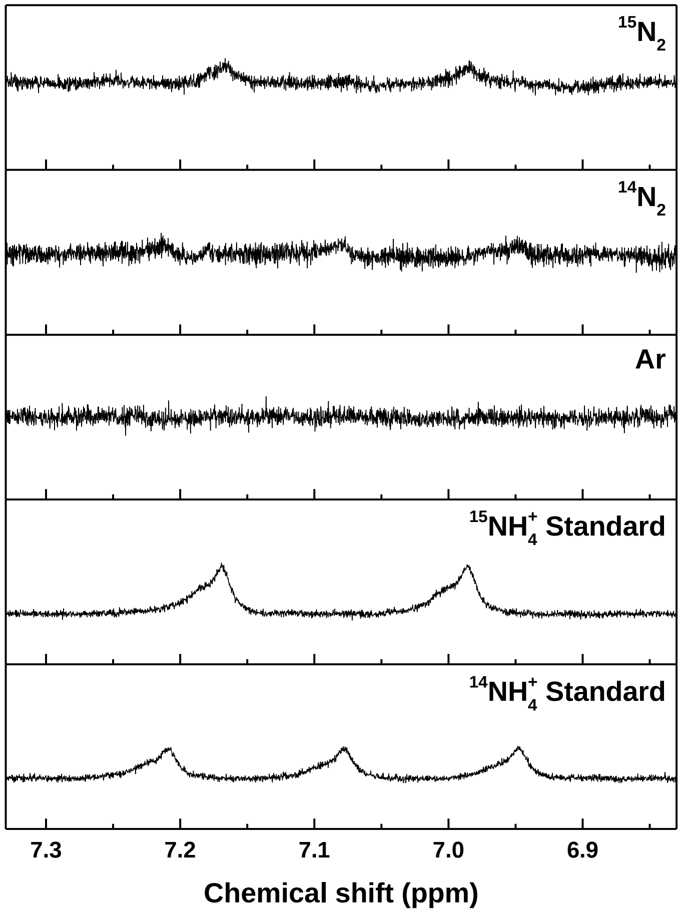

Supplementary Figure 8. ^{15}N isotope labeling experiment. ^{1}H nuclear magnetic resonance (NMR) spectra were obtained for the post-electrolysis 0.1 M PBS electrolytes with $^{15}N_2$, $^{14}N_2$, or Ar as the feeding gas. The chemical shifts in the spectra were calibrated using dimethyl sulphoxide (DMSO) as an internal standard. In the ^{1}H NMR spectra, a doublet coupling for $^{15}NH_4^+$ and a triplet coupling for $^{14}NH_4^+$ were distinguished for the $^{15}N_2$- and $^{14}N_2$-saturated electrolytes after electrolysis, confirming that the NH_3 was produced from the feeding gas. No apparent signals were observed when the electrolyte was bubbled with Ar, indicating a negligible amount of background NH_3 from the catalyst, the electrolyte or the environment, which is consistent with the control experiment using the indophenol blue method in Fig. 2f.

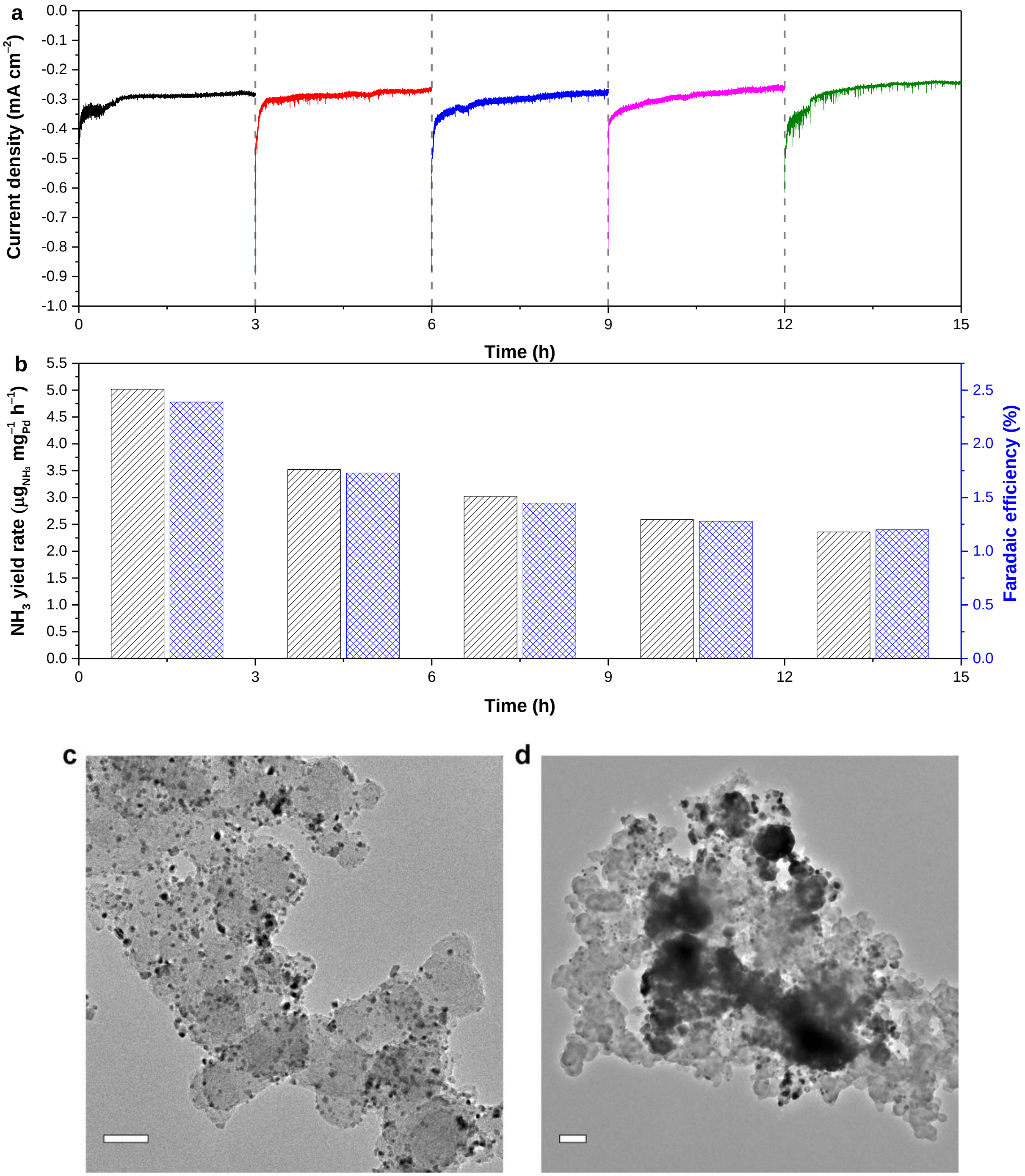

Supplementary Figure 9. Durability measurement. a Stability test of the Pd/C catalyst in N_2-saturated 0.1 M PBS at −0.05 V vs. RHE under consecutive recycling electrolysis. **b** Corresponding NH_3 yield rates and Faradaic efficiencies calculated after each cycle. TEM images of the Pd/C catalyst **c** before and **d** after the stability test. The scale bars are 50 nm in **c** and 100 nm in **d**.

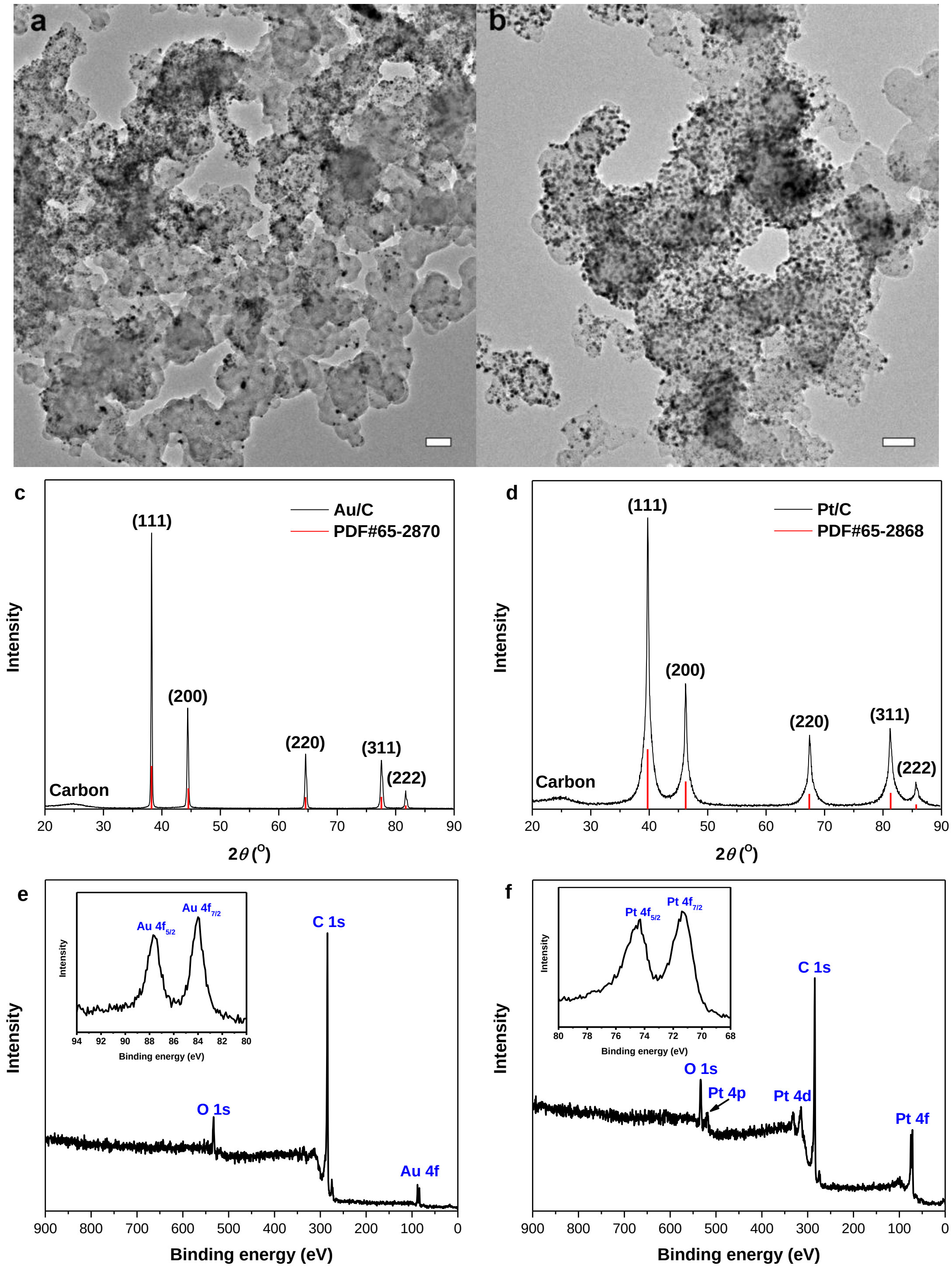

Supplementary Figure 10. Physical characterizations of Au/C and Pt/C catalysts. a, b TEM images, **c, d** XRD patterns, and **e, f** XPS spectra of the **a, c, e** Au/C and **b, d, f** Pt/C catalysts prepared via the polyol reduction method.

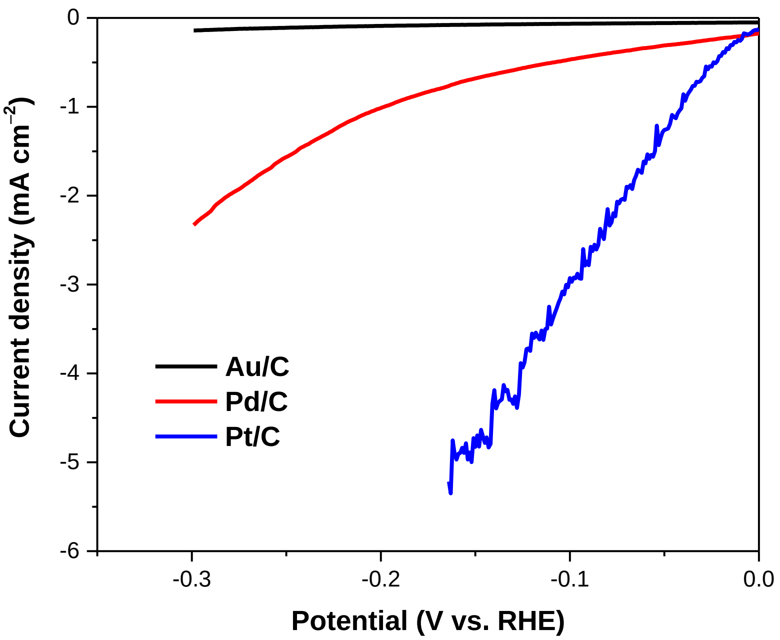

Supplementary Figure 11. Linear sweep voltammograms of the glassy-carbon-supported Au/C, Pd/C, and Pt/C catalysts measured in Ar-saturated 0.1 M PBS at a scan rate of 5 mV s^{-1}.

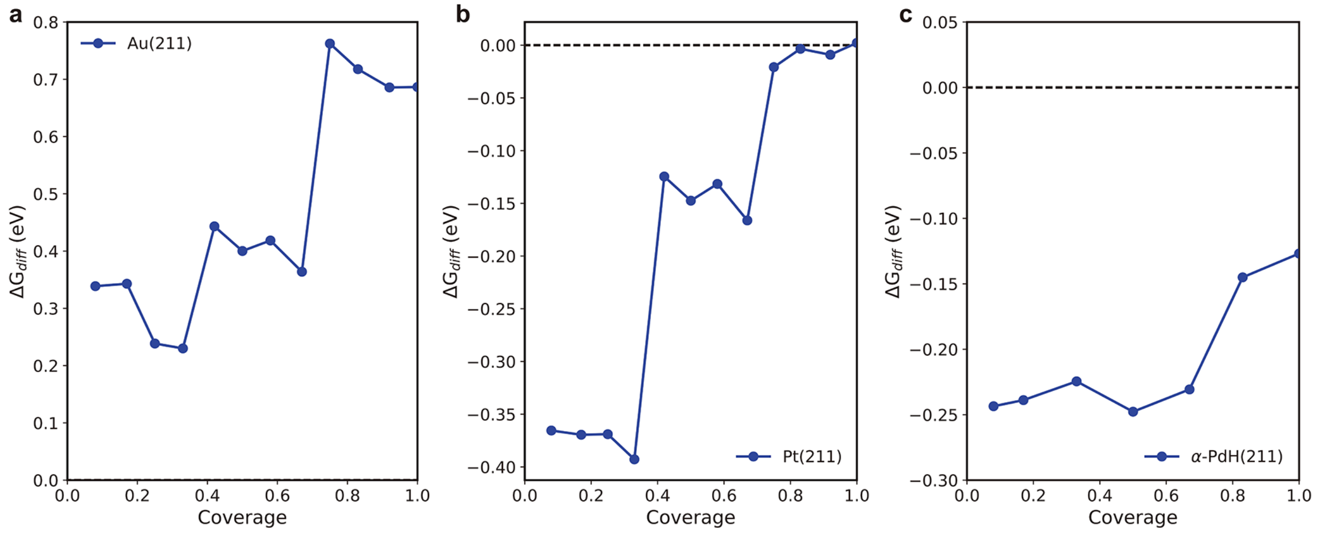

Supplementary Figure 12. Differential adsorption free energy diagram of *H. Differential adsorption free energy diagram of *H was calculated for different *H coverages on the (211) surfaces of **a** Au, **b** Pt, and **c** α-PdH.

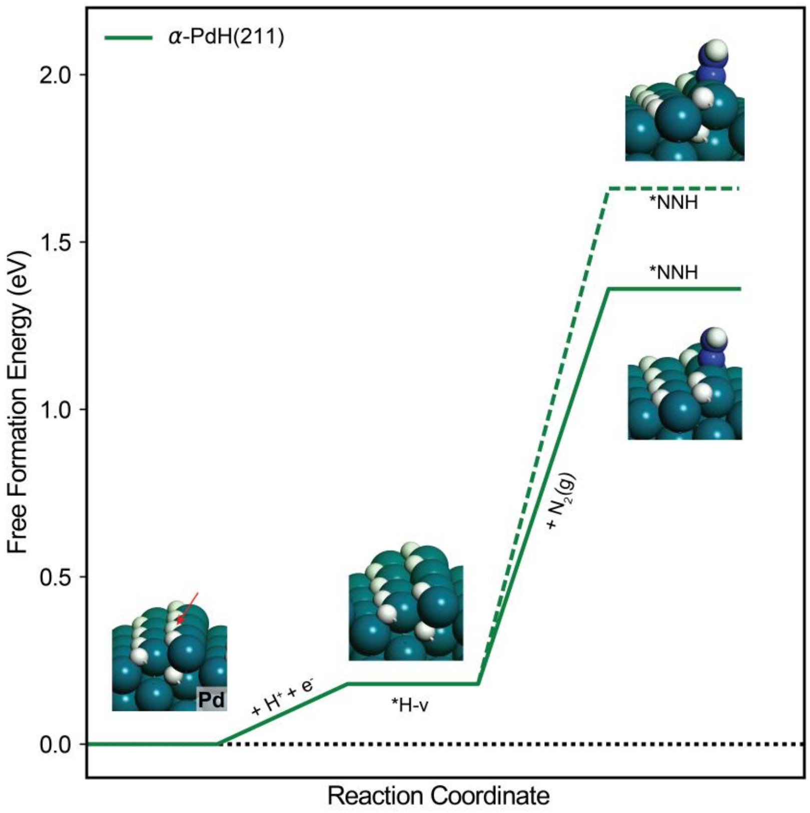

Supplementary Figure 13. Comparison of free energy change for surface hydrogenation of N_2 on α-PdH(211) with (solid line) and without (dashed line) hydride transfer.

Supplementary Table 1. Comparison of the N_2RR performance of the Pd/C catalyst with other catalysts reported to date under ambient conditions (room temperature and atmospheric pressure).

**Catalyst**	**Electrolyte**	**Potential V (vs. RHE)**	**NH_3 yield rate**	**Faradaic efficiency**	**Reference**
**Pd/C**	0.1 M PBS	0.1	4.4 μg mg^{-1}_{Pd} h^{-1}	8.2%	This work
		−0.05	4.9 μg mg^{-1}_{Pd} h^{-1}	2.4%	
**Au nanorod**	0.1 M KOH	−0.2	1.648 μg cm^{-2} h^{-1}	3.88%	*Adv. Mater.* **29**, 1604799 (2017).
**Au cluster/TiO_2**	0.1 M HCl	−0.2	21.4 μg mg^{-1} h^{-1}	8.11%	*Adv. Mater.* **29**, 1606550 (2017).
**a-Au/CeO-RGO**	0.1 M HCl	−0.2	8.3 μg mg^{-1} h^{-1}	10.1%	*Adv. Mater.* **29**, 1700001 (2017).
**PEBCD/C**	0.5 M Li_2SO_4	−0.5	1.58 μg cm^{-2} h^{-1}	2.85%	*J. Am. Chem. Soc.* **139**, 9771−9774 (2017).
**Ru**	2 M KOH	−0.96 (vs. Ag/AgCl)	0.25 μg cm^{-2} h^{-1}	0.92%	*Chem. Commun.* 1673−1674 (2000).
**Pt**	Polymer electrolyte	1.6 V (cell voltage)	11.4 μmol m^{-2} s^{-1}	0.52%	*Sci. Rep.* **3**, 1145 (2013).
**Fe_2O_3/CNT**	$KHCO_3$	−2.0 (vs. Ag/AgCl)	0.22 μg cm^{-2} h^{-1}	<0.05%	*Angew. Chem. Int. Ed.* **56**, 2699−2703 (2017).

Supplementary Table 2. Zero point energy corrections and entropic contributions to the free energies at T = 298.15 K.

	**ZPE (eV)**	**TS (eV)**
**N_2**	0.15	0.59
**H_2**	0.27	0.44
***H**	0.17	0
***N_2H**	0.48	0.15

Supplementary Note 1

(a) Chemicals

Sulfuric acid (H_2SO_4, 98%, TraceMetal grade), sodium hydroxide (NaOH, 98.5%, for analysis), sodium phosphate monobasic monohydrate ($NaH_2PO_4 \cdot H_2O$, 99+%, for analysis), sodium phosphate dibasic heptahydrate ($Na_2HPO_4 \cdot 7H_2O$, 99+%, for analysis), ammonium chloride (NH_4Cl, 99.5+%, certified ACS), trisodium citrate ($C_6H_5Na_3O_7$, anhydrous, 99%), salicylic acid ($C_7H_5NaO_3$, sodium salt, 99+%), sodium nitroferricyanide dihyrate ($C_5FeN_6Na_2O \cdot 2H_2O$, 99+%), sodium hypochlorite (NaClO, 11-15% available chlorine), ethylene glycol ($C_2H_6O_2$, 99.8+%, certified grade), hydrogen peroxide (H_2O_2, 30%, certified ACS), palladium chloride ($PdCl_2$, 99.9%, metals basis), tetrachloroauric acid trihydrate ($HAuCl_4 \cdot 2H_2O$, ACS reagent), hydrogen hexachloroplatinate hydrate ($H_2PtCl_6 \cdot xH_2O$, 99.995%, trace metal basis), ethanol (C_2H_5OH, 88-91%), hydrochloric acid (HCl, 37%, trace metal grade), and *p*-dimethylaminobenzaldehyde ($C_9H_{11}NO$, PDAB, 99+%), deuterium oxide (D_2O, 99.8 atom% D) were purchased from Fisher Scientific. Potassium chloride (KCl, ACS reagent, 99.0−100.5%), Nafion perfluorinated resin solution (5%), ammonium sulfate ($(NH_4)_2SO_4$, 99+%), ammonium-$^{15}N_2$ sulfate ($(^{15}NH_4)_2SO_4$, 98 atom% ^{15}N), and nitrogen-$^{15}N_2$ (98 atom% ^{15}N) were purchased from Sigma-Aldrich. Ultra-high purity 5.0 grade nitrogen was purchased from Airgas. Carbon paper (Spectracarb 2050A-1550), carbon black (Vulcan XC-72), and Nafion 115 membranes were purchased from Fuel Cell Store. Deionized water with the specific resistance of 18.2 MΩ·cm was obtained by reverse osmosis followed by ion exchange and filtration.

(b) Computational Methods

Density functional theory (DFT) calculations are performed using the plane-wave based PWSCF (Quantum-ESPRESSO) program and the Atomic Simulation Environment (ASE)[1]. The ultrasoft Vanderbilt pseudopotential method with Perdew-Burke-Ernzerhof (PBE) exchange-correlation functional is adopted[2–5]. A plane-wave cutoff energy of 500 eV is used. The (211) slabs are built with 4 atomic layers in the [111] direction in rectangular 1×4 supercells with the bottom two [111] layers fixed during structural relaxation. The Monkhorst-Pack scheme is used for sampling the Brillouin zone and k-point grid of 4×2×1 are selected[6]. The vacuum thickness is set to 13 Å in all slab calculations and the dipole correction is applied to decouple the interaction between slabs. During structural optimizations, the residual force

between atoms is converged to a value below 0.02 eV/Å. The free energies of adsorbates are calculated as E_{total} + ZPE − TS_{vib}, where E_{total} is DFT calculated total energy, ZPE is the zero point energy, T is temperature, and S_{vib} is the entropic part from vibrations derived in a harmonic approximation to the potential. The free energies of gas phase molecules are calculated as E_{total} + ZPE − TS, where S is the entropy. We use the free energy of ½H_2 as that of $H^+ + e^-$ by referring the potential to the normal hydrogen electrode[7]. The free energy corrections (ZPE and TS) for adsorbates and molecules are tabulated in **Supplementary Table 2**. The effect of electric potential on the free energy change is included for states that involve electron transfer by using a correction $\Delta G(U) = neU$, where n is number of electrons transferred and U is the electrode potential relative to the reversible hydrogen electrode[7]. We use solvent stabilization energy of 0.1 eV for adsorbate *NNH[8].

Supplementary References

1. Giannozzi, P. et al. QUANTUM ESPRESSO: a modular and open-source software project for quantum simulations of materials. *J. Phys. Condens. Matter* **21**, 395502 (2009).
2. Laasonen, K., Car, R., Lee, C. & Vanderbilt, D. Implementation of ultrasoft pseudopotentials in *ab initio* molecular dynamics. *Phys. Rev. B* **43**, 6796−6799 (1991).
3. Laasonen, K., Pasquarello, A., Car, R., Lee, C. & Vanderbilt, D. Car-Parrinello molecular dynamics with Vanderbilt ultrasoft pseudopotentials. *Phys. Rev. B* **47**, 10142−10153 (1993).
4. Perdew, J. P., Burke, K. & Ernzerhof, M. Generalized gradient approximation made simple. *Phys. Rev. Lett.* **77**, 3865−3868 (1996).
5. Perdew, J. P., Burke, K. & Ernzerhof, M. Generalized gradient approximation made simple. *Phys. Rev. Lett.* **78**, 1396−1396 (1997).
6. Monkhorst, H. J. & Pack, J. D. Special points for Brillouin-zone integrations. *Phys. Rev. B* **13**, 5188−5192 (1976).
7. Nørskov, J. K. et al. Origin of the overpotential for oxygen reduction at a fuel-cell cathode. *J. Phys. Chem. B* **108**, 17886−17892 (2004).
8. Montoya, J. H., Tsai, C., Vojvodic, A. & Nørskov, J. K. The challenge of electrochemical ammonia synthesis: a new perspective on the role of nitrogen scaling relations. *ChemSusChem* **8**, 2180−2186 (2015).
